# Associations between time in range and insulin secretory capacity in Japanese patients with type 2 diabetes

**DOI:** 10.1038/s41598-024-63678-5

**Published:** 2024-06-05

**Authors:** Kenichi Tanaka, Yosuke Okada, Fumi Uemura, Yoshiya Tanaka

**Affiliations:** First Department of Internal Medicine, School of Medicine, University of Occupational and Environmental Health, Japan, 1-1 Iseigaoka, Yahatanishi-ku, Kitakyushu, 807-8555 Japan

**Keywords:** Time in range, C-peptide, Type 2 diabetes, Continuous glucose monitoring, Diabetes, Metabolic disorders

## Abstract

Impaired insulin secretory capacity is associated with high glycemic variability in patients with type 2 diabetes (T2DM). However, there are no existing reports on the association between insulin secretory capacity and time in range (TIR). This retrospective study involved 330 T2DM admitted for diabetes education who underwent intermittently scanned continuous glucose monitoring (isCGM) and had their fasting serum C-peptide immunoreactivity (S-CPR) measured within 5 days of admission. The baseline characteristics were as follows: age, 60.2 years; glycated hemoglobin (HbA1c), 9.2%; S-CPR, 2.2 ng/mL; S-CPR index (S-CPR [ng/mL]/fasting plasma glucose [mg/dL] × 100), 1.6; and TIR, 60.3%. TIR correlated significantly with the S-CPR index, which was confirmed by multivariate analysis that included various factors such as HbA1c. Receiver operating characteristic (ROC) analysis showed that 1.88 was the optimal S-CPR index level to predict TIR ≥ 70%. In addition to HbA1c and biguanide use, the S-CPR index was a significant factor associated with TIR > 70%. S-CPR index values of ≥ 1.88 also correlated significantly with TIR > 70%. In conclusion, insulin secretory capacity is associated with TIR in Japanese T2DM, suggesting that the S-CPR index might be a potentially useful biomarker insulin secretory capacity, in association with TIR.

*Trial registration* UMIN0000254333.

## Introduction

Diabetes is a metabolic disease characterized by chronic hyperglycemia caused by diminished insulin sensitivity in peripheral tissues as well as relatively insufficient insulin action due to impaired insulin secretion from pancreatic β cells. The pancreatic β cell mass decreases with progression from non-diabetes to diabetes^1^, and β cell function is decreased by 50% or more at the onset of diabetes^2^.

**Figure 1:**
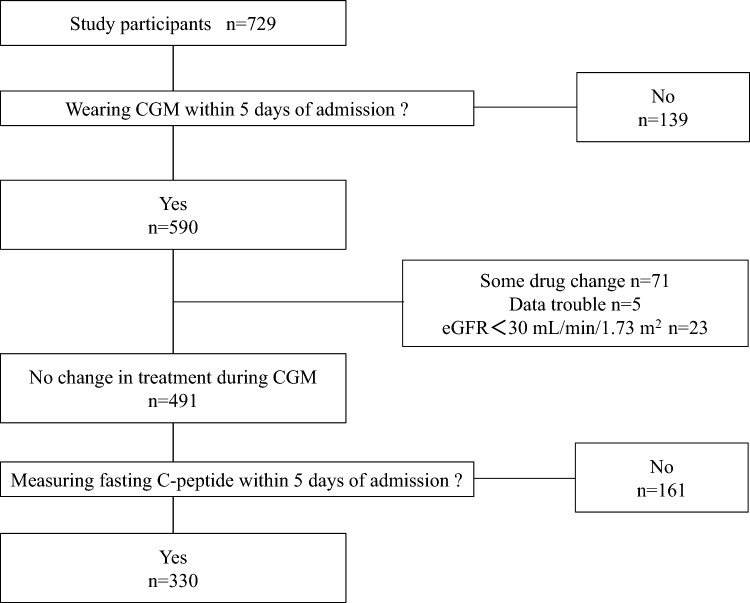
Protocol used in the present study for recruitment of 330 patients with type 2 diabetes.

The prevalence of obesity in Japanese people is relatively lower than in Europeans and Americans; the insulin secretory capacity during the stage of normal glucose tolerance is lower in Japanese people^3^. Furthermore, the secretory capacity of pancreatic β cells in Japanese patients is lower than in European and American patients due to the fact that the pathology of Japanese patients with type 2 diabetes is affected by early-phase impairment of insulin secretion^4,5^. Thus, impaired insulin secretion is greatly associated with the pathology of diabetes in Asians, including Japanese people (Fig. 1).

Oxidative stress increases with higher glycemic variability^6,7^. The continuous glucose monitoring (CGM) system is used to monitor average glucose level, glycemic variability, hypoglycemia, and related metrics. Oxidative stress in patients with type 2 diabetes is associated with rapid glycemic variability; represented by the mean amplitude of glycemic excursions (MAGE), as measured by the CGM^8^. That MAGE is associated with cardiovascular events^9^ has highlighted the importance of lessening glycemic variability with glucose-lowering treatments.

In recent years, the concept of time in range (TIR) derived from the CGM has been internationally standardized as an index of glycemic control. TIR represents the percentage of time spent within 24 h at interstitial glucose levels ranging from 70 to 180 mg/dL, and TIR of > 70% is recommended as a target of type 2 diabetes management to prevent vascular complications^10^.

A study of patients with type 1 diabetes using CGM reported that preserved insulin secretion, as reflected by C-peptide concentration (C-peptide: 10–200 pmol/L [0.03–0.67 ng/mL]) was associated with a lower incidence of hypoglycemic events and a decrease in glycemic variability^11^. Furthermore, low C-peptide levels are an independent factor for high glycemic variability (coefficient of variation) in type 2 diabetes^12,13^. However, to our knowledge, there are no studies on the relationship between insulin secretory capacity and TIR in type 2 diabetes. In this study, we investigated the association between insulin secretory capacity and TIR in Japanese patients with type 2 diabetes and impaired insulin secretory capacity.

## Results

### Patient demographics

The baseline characteristics of the study patients are summarized in Table 1. The study included 330 patients with type 2 diabetes, with a mean disease duration of 10.4 years and mean HbA1c level of 9.2%. The mean S-CPR and S-CPR index were 2.2 ng/mL and 1.6, respectively. As for the CGM parameters, the AG level was 174.3 mg/dL; TIR was 60.3%, and TAR was 38.8%. These values indicated poor glycemic control.

**Table 1:** Baseline characteristics of type 2 diabetes patients.

n	330
Age (years)	60.2 ± 14.3
Men (%)	193 (58.5)
Duration of diabetes (year)	10.4 ± 9.7
Body mass index (kg/m^2^)	26.7 ± 5.7
EGFR (mL/min/1.73 m^2^)	79.7 ± 29.1
FPG (mg/dL)	153.6 ± 45.9
HbA1c (%)	9.2 ± 1.8
S-CPR (ng/mL)	2.2 ± 1.2
S-CPR index	1.6 ± 0.9
Diabetes therapy	
No medication (%)	87 (26.4)
DPP-4 inhibitor (%)	151 (45.8)
Sulfonylurea (%)	78 (23.6)
Glinide (%)	7 (2.1)
Biguanide (%)	91 (27.6)
Thiazolidine (%)	40 (12.1)
α-glucosidase inhibitor (%)	25 (7.6)
SGLT-2 inhibitor (%)	22 (6.7)
Insulin (%)	75 (22.7)
GLP-1 receptor agonist (%)	7 (2.1)
Diabetic vascular complications	
Retinopathy (%)	117 (35.5)
Nephropathy (%)	102 (30.9)
Neuropathy (%)	168 (50.9)
Macro-angiopathy (%)	51 (15.5)
AG (mg/dL)	174.3 ± 46.1
SD (mg/dL)	38.2 ± 13.8
%CV (%)	22.3 ± 7.2
MAGE (mg/dL)	94.6 ± 34.7
Maximum glucose (mg/dL)	262.3 ± 60.0
Minimum glucose (mg/dL)	113.2 ± 38.7
TAR (%)	38.8 ± 30.4
TIR (%)	60.3 ± 30.0
TBR (%)	0.8 ± 4.3

Data are mean ± standard deviation, or n (%).

EGFR, estimated glomerular filtration rate; FPG, fasting plasma glucose; HbA1c, glycated hemoglobin; S-CPR, serum C peptide immunoreactivity; DPP-4, dipeptidyl peptidase-4; SGLT-2, Sodium-glucose cotransporter 2; GLP-1, glucagon-like peptide-1; AG, average glucose; SD, standard deviation of average glucose; %CV, % coefficient variation; MAGE, mean amplitude of glycemic excursions; TAR, time above range; TIR, time in range; TBR, time below range.

### Relationship between S-CPR index levels and baseline characteristics

Table 2 shows the correlation coefficients between S-CPR index levels and the baseline characteristics of patients with type 2 diabetes. The S-CPR index levels correlated negatively with age and disease duration but positively with BMI. In addition, S-CPR index levels correlated negatively with HbA1c (a marker of AG levels) and AG, and also with SD and MAGE (markers of glycemic variability). Furthermore, the S-CPR index correlated significantly with TIR and negatively with TAR.

**Table 2:** Correlation coefficients between S-CPR-index levels and the baseline characteristics of type 2 diabetes patients.

	Type 2 diabetes patients	
	r	*P*-value
Age	− 0.257	< 0.001
Duration of diabetes	− 0.378	< 0.001
Body mass index	0.459	< 0.001
EGFR	− 0.046	0.625
FPG	− 0.313	< 0.001
HbA1c	− 0.209	< 0.001
AG	− 0.317	< 0.001
SD	− 0.252	< 0.001
%CV	− 0.033	0.548
MAGE	− 0.207	< 0.001
Maximum glucose	− 0.313	< 0.001
Minimum glucose	− 0.204	< 0.001
TAR	− 0.325	< 0.001
TIR	0.336	< 0.001
TBR	− 0.013	0.814

Data are results of Pearson’s correlation analysis for normally distributed variables and Spearman rank correlation for variables with skewed distribution. Abbreviations as in Table 1.

### Characteristics of patients with high S-CPR index levels

**Figure 2:**
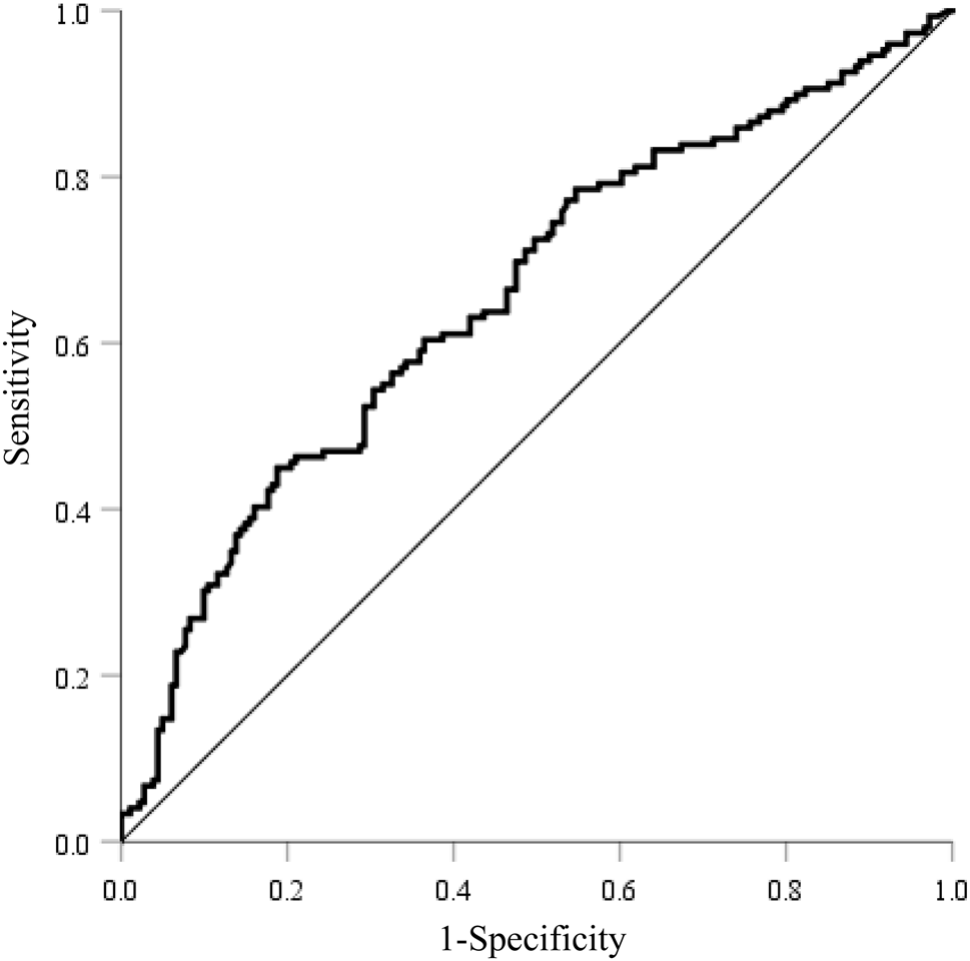
The ROC curves of S-CPR index level for predicting TIR > 70% in patients with type 2 diabetes. ROC, receiver operating characteristic; S-CPR, serum C peptide immunoreactivity; TIR, time in range.

The ROC curve analysis identified a threshold of 1.88 as the optimal S-CPR index level for predicting TIR > 70% in patients with type 2 diabetes. This threshold exhibited a sensitivity of 45% and specificity of 81%, with an area under the ROC curve (AUC) of 0.655 (95%CI 0.596–0.715) (Fig. 2). Using this threshold value, we divided the patients into two groups: those with low S-CPR index levels (S-CPR index < 1.88) and those with high S-CPR index levels (S-CPR index ≥ 1.88), and their characteristics were compared (Table 3). Patients with high S-CPR index levels were significantly younger and had shorter disease duration and higher BMI. Furthermore, HbA1c levels were significantly lower in the high S-CPR index group. For the CGM parameters, patients of the high S-CPR index group had significantly lower AG and TAR but higher TIR, compared with those of the low S-CPR index group. Furthermore, various markers of glycemic variability, such as SD and MAGE, were significantly lower in the patients of the high S-CPR index group.

**Table 3:** Comparison between type 2 diabetes patients with low and high S-CPR index levels.

	Low S-CPR index	High S-CPR index	*P*
N	227	103	
Age (years)	61.8 ± 13.9	56.6 ± 14.5	0.002
Men (%)	129 (56.8)	64 (62.1)	0.365
Duration of diabetes (year)	11.9 ± 10.3	7.2 ± 7.4	< 0.001
Body mass index (kg/m^2^)	25.4 ± 5.0	29.7 ± 6.2	< 0.001
EGFR (mL/min/1.73 m^2^)	82.8 ± 31.3	72.9 ± 22.4	0.004
FPG (mg/dL)	162.2 ± 46.1	136.1 ± 38.7	< 0.001
HbA1c (%)	9.5 ± 1.8	8.7 ± 1.6	< 0.001
S-CPR (ng/mL)	1.71 ± 0.76	3.40 ± 1.03	< 0.001
CPR index	1.10 ± 0.43	2.62 ± 0.61	< 0.001
AG (mg/dL)	182.5 ± 47.6	156.2 ± 37.0	< 0.001
SD (mg/dL)	39.6 ± 13.7	34.9 ± 13.6	0.004
%CV (%)	22.2 ± 7.3	22.4 ± 7.2	0.811
MAGE (mg/dL)	97.6 ± 33.5	88.1 ± 36.7	0.021
Maximum glucose (mg/dL)	272. 7 ± 60.4	239.4 ± 52.5	< 0.001
Minimum glucose (mg/dL)	118.6 ± 41.9	101.3 ± 27.2	< 0.001
TAR (%)	44.6 ± 31.0	26.2 ± 24.9	< 0.001
TIR (%)	54.6 ± 30.5	72.9 ± 24.7	< 0.001
TBR (%)	0.8 ± 4.5	1.0 ± 3.9	0.747

Data are mean ± standard deviation, or n (%).

*P* values by the Student’s t-test for normally distributed data and Wilcoxon rank-sum test for data with skewed distribution. Categorical values were tested by − χ2 test. *P* values are for differences between the two groups. Abbreviations as in Table 1.

### Association between TIR and S-CPR index

Finally, we investigated the association between TIR and S-CPR index. TIR correlated significantly with the S-CPR index, which was confirmed by multivariate analysis that included various other factors such as HbA1c (Table 4). Table 5 shows the results of logistic analysis performed to identify factors associated with TIR > 70%. In addition to HbA1c and biguanide use, the S-CPR index was a significant factor associated with TIR > 70%. Furthermore, the S-CPR index remained significantly associated with TIR > 70% (odds ratio: 2.198; 95% CI 1.247–3.875; *P* = 0.006) when “S-CPR index ≥ 1.88” was used instead of the S-CPR index.

**Table 4:** Multivariate linear regression analysis with TIR as the dependent variables in type 2 diabetes patients.

Variables	Univariate		Multivariate	
	β (95% CI)	*P*-value	β (95% CI)	*P*-value
Age	− 0.096 (− 0.429, 0.026)	0.082	− 0.165 (− 0.621, − 0.072)	0.013
Men	0.043 (− 4.008, 9.185)	0.441		
Duration of diabetes	− 0.112 (− 0.679, − 0.012)	0.008		
Body mass index	0.168 (0.321, 1.443)	0.002		
EGFR	− 0.142 (− 0.257, − 0.036)	0.010	− 0.160 (− 0.286, − 0.044)	0.008
HbA1c	− 0.455 (− 9.149, − 5.942)	0.006	− 0.421 (− 8.655, − 5.374)	< 0.001
S-CPR index	0.331 (7.951, 15.115)	< 0.001	0.120 (0.037, 8.350)	0.048
Retinopathy	− 0.159 (− 16.642, − 3.218)	0.004		
Neuropathy	− 0.160 (− 15.991, − 3.143)	0.004		
Biguanide	0.150 (2.873, 17.267)	0.006	0.142 (3.117, 15.858)	0.004
Glinide	− 0.158 (− 55.105, − 10.516)	0.004	− 0.112 (− 42.350, − 4.079)	0.018
			R^2^ = 0.314	
			*P* < 0.001	

Age, sex (men), duration of diabetes and factors with *P* < 0.05 were entered in this multivariate linear regression analysis.

**Table 5:** Multiple logistic regression analyses of variables contributing to TIR > 70% in type 2 diabetes patients.

	Univariate logistic regression analysis		Multiple logistic regression analysis	
	*P*	Odds ratio (95% CI)	*P*	Odds ratio (95% CI)
Age	0.013	0.981 (0.965, 0.996)		
Men	0.677	1.098 (0.707, 1.706)		
Duration of diabetes	0.164	0.984 (0.962, 1.007)		
Body mass index	0.001	1.072 (1.029, 1.116)		
HbA1c	< 0.001	0.703 (0.610, 0.809)	< 0.001	0.674 (0.571, 0.794)
S-CPR index	< 0.001	1.940 (1.468, 2.563)	0.046	1.430 (1.007, 2.030)
Retinopathy	0.026	0.592 (0.373, 0.940)		
Biguanide	0.001	2.357 (1.438, 3.862)	0.003	2.323 (1.328, 4.063)
S-CPR-index ≧ 1.88^†^	< 0.001	3.291 (2.022, 5.357)	0.006	2.198 (1.247, 3.875)

Age, sex (men), duration of diabetes and factors with *P* < 0.05 on univariate logistic regression were included in this multiple logistic regression.

^†^S-CPR-index ≧ 1.88 as a variable instead of S-CPR index in another multiple logistic regression analysis model.

## Discussion

Our study demonstrated a significant association between S-CPR index and TIR in Japanese patients with type 2 diabetes, demonstrating for the first time that TIR increases with preserved insulin secretory capacity. The results also showed that a S-CPR index of 1.88 (odds ratio: 2.2) achieves TIR > 70% in Japanese patients with type 2 diabetes.

Recent CGM-based studies have shown that preserved insulin secretion is associated with low incidence of hypoglycemic episodes and decrease in glycemic variability in patients with type 1 diabetes^11^. However, no consensus has been reached regarding patients with type 2 diabetes. Although low C-peptide levels are an independent factor for high glycemic variability (coefficient of variation)^12,13^, C-peptide levels are associated with CV in patients with type 2 diabetes on insulin therapy but not in patients with type 2 diabetes untreated with insulin^14^. These differences in views may be attributable to the differences in sample size and insulin secretory capacity among reports.

This is the first study that demonstrated the association of S-CPR index with TIR in patients with type 2 diabetes. TIR was higher in patients with high S-CPR index levels (≥ 1.88) than in those with low S-CPR index levels (< 1.88), and multivariate analysis also showed that the S-CPR index correlated positively with TIR. Furthermore, the two markers used in this study for glycemic variability (SD and MAGE) were significantly lower in patients with high S-CPR index levels. The S-CPR index was not associated with %CV. A possible reason for this is that the effects of these indicators might have been canceled because the equations used for calculating both indicators include fasting plasma glucose levels. Hypoglycemia was also not associated with the S-CPR index. This may be attributable to the low TBR due to the high insulin secretory capacity. With regard to the use of antidiabetic agents, metformin use was associated with TIR, whereas glinide use was negatively associated with TIR. It is possible that these findings might have been affected by the fact that metformin is more likely to be used in patients with insulin resistance and that glinide is more likely to be used in patients with impaired insulin secretion. It is also possible that confounding factors that were not measured in the study might have contributed to these findings. Further studies are needed to dissect the impact of treatment.

In 2019, TIR was internationally standardized as a metric for glycemic control derived from CGM^10^. Furthermore, maintaining TIR > 70% was recommended as a target for managing type 2 diabetes to prevent microangiopathies, based on research demonstrating that a TIR of 70% corresponds to an HbA1c level of approximately 7%^15^. Our study showed that the cut-off value of S-CPR index for achieving TIR > 70% was 1.88, suggesting that the S-CPR index might also be a useful biomarker for glycemic control, in association with TIR. In addition, recent clinical studies have shown that low TIR is associated with the onset and progression of microvascular complications^16^ and with increased risks of all-cause mortality and cardiovascular disease (CVD) mortality^17^ in patients with diabetes. In a series of studies based on analysis of CGM data of 999 Japanese patients with type 2 diabetes, we have reported recently that glycemic variability and hypoglycemia correlated with the onset and progression of micro- ^18^ and macro-vascular complications^19,20^. Thus, maintaining high TIR is extremely important with respect to the prevention of diabetic complications and survival prognosis. The results of the present study highlighting the association between TIR and the S-CPR index reaffirmed the importance of early diagnosis and therapeutic interventions for diabetes to prevent impairment of insulin secretory capacity.

In addition to the fact that β-cell secretory capacity is lower in Japanese people than in Europeans and Americans^4,5^, a recent study has identified new genetic loci associated with type 2 diabetes in East Asians^21^. Furthermore, a family history of diabetes is associated with impaired β-cell function in Japanese and Chinese people^22,23^. Heredity-related impairment of insulin secretory capacity is an important pathological feature in East Asians. We were unable to investigate the association of the family history with insulin secretory capacity or TIR since we could not determine the presence or absence of a family history of diabetes. Such association is an issue for future studies.

The present study has several limitations. First, the study participants were only Japanese patients; therefore, it is uncertain whether the findings are applicable to other populations, especially those with higher insulin secretory capacity. Second, the current study was only based on data obtained from hospitalized patients. During hospitalization, it was confirmed that C-peptide levels were measured in the early morning fasting state after fasting for at least 12 h. Meanwhile, inpatient dietary management may improve daily CGM data, such as TIR. However, we took care to reduce as many impact factors related to hospitalization as possible, by for example including only patients who initiated CGM within 5 days of admission and excluding those who changed the doses or types of hypoglycemic agents during the study. Third, we did not investigate the family history of diabetes, as discussed above. This might have affected the data of insulin secretory capacity. Thus, further randomized trials are needed to determine the impact of these factors.

In conclusion, our study is the first to demonstrate that insulin secretory capacity is associated with TIR in Japanese patients with type 2 diabetes, showing that TIR increases with preserved insulin secretory capacity. Furthermore, the results identified S-CPR index level of ≥ 1.88 to be associated with achieving TIR > 70% in patients with type 2 diabetes, suggesting that this index is a potentially useful biomarker of glycemic control, in association with TIR.

## Methods

### Patients

The study patients were selected from patients with type 2 diabetes admitted to the Hospital of the University of Occupational and Environmental Health, Japan, or its affiliated hospitals, for education program on glycemic control and diabetes management between April 2010 and March 2020, using the following criteria: *1)* patients who underwent intermittently scanned continuous glucose monitoring (isCGM) within 5 days of admission, *2)* patients who showed no change in treatment after isCGM monitoring, and *3)* patients who underwent measurement of fasting serum C-peptide immunoreactivity (S-CPR) within 5 days of admission. After excluding patients who changed the doses or types of hypoglycemic agents after wearing a isCGM device and those with renal dysfunction (estimated glomerular filtration rate [eGFR] of < 30 mL/min/1.73 m^2^), we collected and analyzed the isCGM data of the remaining 330 patients (Fig. 1).

Based on the guidelines of the Japan Diabetes Society, energy intake was set as follows: total energy intake was set at 25–30 kcal/kg of ideal body weight, with the target composition of energy intake comprising 50–60% from carbohydrates, 20% or lower from proteins, and the remaining percentage from fat. The contents of exercise therapy were unchanged, and efforts were made to maintain the intensity of exercise constant.

This study was conducted in accordance with the Declaration of Helsinki and the current ethical codes. The study protocol was approved by the ethics committee of the University of Occupational and Environmental Health, Japan (Approval No.*UOEHCRB21-105) and its affiliated hospitals (University Medical Information Network [UMIN] ID: UMIN0000254333). Informed consent was obtained from all participants.

### Study design

This was a retrospective study. The following data recorded at admission were collected from the medical records: age, sex, arterial blood pressure, body mass index (BMI), duration of diabetes, presence of diabetic microangiopathies or macroangiopathies, presence of hypertension, presence of dyslipidemia, antidiabetic drug use, antihypertensive drug use, and antilipidemic drug use. The levels of fasting plasma glucose, glycated hemoglobin (HbA1c), and serum C-peptide (S-CPR) were measured within 5 days of admission.

### Continuous glucose monitoring system

The isCGM devices used in this study were the Gold™ (CGMS System Gold, Medtronic Inc., Fridley, MN) and iPro™2 (Medtronic MiniMed Inc., Northridge, CA). These devices continuously measure interstitial glucose levels within a range of 40–400 mg/dL. The sensor placed in the subcutaneous tissue converts the interstitial glucose levels into electrical signals and records measurements every 5 min, up to a maximum of 288 measurements per day. The isCGM device was calibrated before each of the three meals and at bedtime using data from a self-monitored blood glucose device (MEDISAFE MINI; Terumo, Inc.).

The isCGM data consisted of 288 measurements recorded from 00:00 to 24:00 on the third day of wearing the device. The analyzed isCGM parameters included average glucose (AG), standard deviation of average glucose (SD), % coefficient variation (%CV), TIR, time above range (TAR, defined as the percent time with glucose levels above 180 mg/dL), and time below range (TBR, defined as the percent time with glucose level less than 70 mg/dL).

### Biochemical and clinical measurements

HbA1c levels (%) were measured using a high-performance liquid chromatography method with a Tosoh HLC-723 G8 analyzer (Tosoh Co., Kyoto, Japan) and expressed in National Glycohemoglobin Standardization Program (NGSP) equivalent values calculated by the following equation: HbA1c (NGSP) = HbA1c (Japan Diabetes Society [JDS]) (%) + 0.4%^24^.

The following formula was used to calculate serum CPR index: S-CPR index = S-CPR (ng/mL)/FPG (mg/dL) × 100. S-CPR levels were measured using chemiluminescence immunoassay (LSI Medience Co., Tokyo, Japan).

### Endpoints

The primary endpoint was the relationship between TIR and S-CPR-index levels.

### Statistical analysis

Data are expressed as means ± standard deviation. Data distribution was determined using the Shapiro–Wilk test. Categorical values were tested by the − χ^2^ test. Correlation analyses between S-CPR index levels and the baseline characteristics of patients were performed using Pearson’s correlation analysis for normally distributed variables and Spearman’s correlation analysis for variables with skewed distribution. Comparison of the characteristics of type 2 diabetes patients with low and high S-CPR index levels was performed using the Student’s t-test for normally distributed data and Wilcoxon rank-sum test for data with skewed distribution. To investigate the predictive value of S-CPR index levels for TIR > 70%, receiver operating characteristic (ROC) curves were plotted to determine the optimal cut-off value of S-CPR index level. In addition, to investigate the association between TIR and S-CPR index, we performed multivariate linear regression analysis and multivariate logistic regression analysis. These two analyses were performed with the following independent variables: age, sex (males), and factors identified with a *P* value of < 0.05 on univariate analysis after exclusion of factors showing multicollinearity on Spearman’s correlation analysis. In the logistic analysis, data were expressed as odds ratios (OR) with 95% confidence intervals (95%CI). All statistical analyses were carried out using SPSS version 25.0 (SPSS Inc., Chicago, IL). A *P* value less than 0.05 was considered to denote statistical significance.

## Data Availability

All data generated or analyzed during this study are included in the published article.
